# Clinical characteristics and genetic analysis of children with Omicron BF.7.14 type novel coronavirus-related acute necrotizing encephalopathy

**DOI:** 10.3389/fneur.2024.1365299

**Published:** 2024-05-30

**Authors:** Jianzhao Zhang, Jing Sun, Dongqing Li, Hua Xie, Shaofang Shangguan, Yangyang Ge, Hui Jiao, Caihui Ma, Zhao Liu, Linqing Zhao, Jian Yang

**Affiliations:** ^1^Neurology Department of Children Hospital Affiliated to Capital Institute of Pediatrics, Beijing, China; ^2^Genetics Research Department Affiliated Capital Institute of Pediatrics, Beijing, China; ^3^Virology Research Department Affiliated Capital Institute of Pediatrics, Beijing, China

**Keywords:** acute necrotizing encephalopathy, CRMP2, inheritance, thalamus, novel coronavirus

## Abstract

**Background:**

To explore the clinical characteristics, etiological factors, and clinical-related genetic variant of children with acute necrotizing encephalopathy (ANE) related to the Omicron BF.7.14 novel coronavirus.

**Methods:**

Genomic variations were detected through whole exome sequencing. Additionally, we summarized the clinical data to explore the inheritance patterns associated with novel coronavirus-related ANE.

**Results:**

This study included four patients (2 males and 2 females) with an average age of 2.78 ± 1.93 years. All the patients had prodromal symptoms of Omicron BF.7.14 virus infection, and exhibited symptoms such as altered consciousness, seizures and cognitive/language disturbances. Cranial MRI scans revealed damage to the thalamus, basal ganglia and brainstem. The cerebrospinal fluid (CSF) cell counts were nearly normal, but protein level in CSF increased significantly. Genetic analysis revealed a novel truncated variant of CRMP2 gene in one patient who suffered more severe coma score and prognosis and dead in the later stages. All children exhibited a decrease in the absolute count of T lymphocytes, helper T cells, suppressor T cells, and NK cells to varying degrees. Furthermore, levels of cytokines, including IL-1 β, IL-5, IL-6 and IL-8 were significantly elevated in the CSF, especially in patient with truncated variant of CRMP2 gene.

**Conclusion:**

The Omicron BF.7.14 type novel coronavirus can lead to ANE, characterized by T cell immunosuppression and a significant increase in cytokine levels in the CSF. The truncated variation of CRMP2 gene may affect the prognosis of ANE by affecting the migration of cerebral T cells.

## Introduction

Acute necrotizing encephalopathy (ANE) is a rare form of acute encephalopathy. During the outbreak of influenza virus, the incidence rate of ANE in children is about 12.83/100 million (1). Common viruses known to cause ANE include the influenza virus, human herpesvirus 6, rotavirus, and the novel coronavirus, which have several reports in the past 2 years (2, 3). Besides viral infections acting as external triggers, genetic susceptibility also plays as a significant role in development of the disease. In 2003, Neilson et al. (4) discovered that familial or recurrent ANE is autosomal dominant inheritance, and reported the first case of familial or recurrent ANE associated with a missense variant in the RANBP2 gene in 2009 (5). Shinohara et al. (6) identified variant in the CPT II (Carnitine Palmitoyltransferase II) gene variant in children with ANE, and Kobayashi et al. also found CPT II gene variant in adult ANE patients (7). RANBP2 has many functions within a cell that could potentially be affected by the variant, resulting in this disease, including interactions with mitochondria, metabolism, and nuclear signaling, with potential effects on viral invasion directly. Variant in the CPT II gene participate in the pathogenesis of ANE by reducing enzyme activity.

Although variant in the RANBP2 and CPT II gene have been reported in ANE, most patients remain without a clearly identified pathogenic gene. Previous studies have also not yet identified novel coronavirus subtypes that cause ANE. Consequently, this study conducted a retrospectively analysis of the clinical manifestations, immunological profiles and genetic findings in of 4 children with ANE following infection with the Omicron BF.7.14 variant of the novel coronavirus. The aim was to elucidate the genetic underpinnings and susceptibility genes associated with novel coronavirus infection-induced ANE. This study aims to provide valuable insights into the pathogenesis of novel coronavirus associated acute necrotizing encephalopathy (ANE) in children.

## Materials and methods

### Participants

In this retrospective study, according to the inclusion criteria, we reported four patients diagnosed with ANE from the Children’s Hospital affiliated with the Capital Institute of Pediatrics during the COVID-19 epidemic period, spanning from November 2022 to February 2023.

The diagnostic criteria for ANE include the following (8): (1) Acute encephalopathy that manifests after acute viral infection, rapidly leading to seizures and consciousness disorders; (2) The cell count in CSF examination is normal, with a typical elevation in cerebrospinal fluid protein levels exceeding 0.45 g/L (normal values ranging from 0.20 to 0.40 g/L); (3) Imaging examination revealed symmetrical and multifocal brain damage, involving the bilateral thalamus (100%), upper brainstem tegmentum, periventricular white matter, cerebellar medulla, internal capsule, putamen, while not involving other areas of the central nervous system; (4) Increased levels of serum transaminases, while blood ammonia and blood glucose levels within the normal range; (5) Exclusion of similar diseases: Diseases that require differential diagnosis based on clinical manifestations include the majority of bacterial and viral infections, fulminant hepatitis, toxic shock, hemolytic uremic syndrome, Reye syndrome, heatstroke, and encephalopathy syndrome; Diseases that require differential diagnosis based on imaging examination results include subacute necrotic encephalopathy, glutaraldehyde, infantile striatal necrosis, Wernicke’s encephalopathy, CO poisoning, acute disseminated encephalomyelitis, acute hemorrhagic encephalopathy, as well as other types of encephalitis, vasculitis, and arteriovenous infarction. All enrolled children underwent genetic testing.

This study was approved by the Ethics Committee of the Capital Institute of Pediatrics, with the ethics number SHERLLM2023061.

### Methods

#### Establish a clinical data database

Collect the following information: (1) general information, such as age, gender, etc.; (2) Clinical features, including consciousness disorders, seizures, language disorders, paralysis, and more; (3) Auxiliary examinations, encompassing serum lymphocytes, cerebrospinal fluid routine, cerebrospinal fluid cytokines, cranial imaging, peripheral blood whole exon data, and so on. The follow-up period is from 6 months to one and a half years, using phone call or hospital visits. The cranial magnetic resonance imaging of all patients was completed within 1–3 days of the onset of central nervous system symptoms, and no enhanced MRI was performed.

#### Etiological detection and virus sequencing

All children underwent testing for COVID-19, and the results confirmed the presence of COVID-19 Omicron BF.7.14 using next-generation sequencing. Testing for other bacteria and viruses yielded negative results.

#### Detection of serum immune cells and CSF cytokines

All samples were collected during the acute phase of the disease, prior to the administration of immune therapy.

#### Genetic testing

2 mL of peripheral venous blood from each child and their parents were collected using anticoagulant blood collection vessels, and peripheral blood DNA was extracted using the QIAamp DNA Blood Midi Kit (QIAGEN, Valencia, CA) kit. Whole exome Conduct family-wide exon sequencing experiment and raw data analysis were performed using standard according to established laboratory protocols. Perform data analysis and interpretation. For rare (<1% MAF in genomAD database), deleterious (predicted by more than two software programs) and clinical-related genetic point mutation variant, landing polymerase chain reaction combined with Sanger sequencing was used to verify the variant inheritance in mutation variant sites of the children and their parents (2 pairs of different primers were designed for each mutation variant site to avoid false positives caused by one-way amplification). Complete pathogenicity assessment according to the guidelines of the American College of Medical Genetics and Genomics (ACMG) (9).

## Results

### Clinical characteristics of ANE children

Among the four children diagnosed with ANE, there were two males and two females, with an average age of 2.78 ± 1.93 years. Apart from one case with a history of febrile seizures, the other three cases were previously in good health. All patients exhibited precursor symptoms of viral infection, along with symptoms of consciousness disturbances, seizures, cognitive/language disturbances, and brain damage. Epilepsy manifests as cluster seizures and status epilepticus. Cranial MRI revealed damage to areas including the thalamus, basal ganglia, and brainstem. The count of white blood cells in the CSF was generally within the normal range, while CSF protein levels varied. Patient P1 had a severe coma score and prognosis, resulting in death within 3 weeks. The remaining patients showed improvement and were subsequently discharged. P1 has a variant in the CRMP2 gene and is the only case of death, which is one of this study highlights. Therefore, the clinical and immune characteristics of the patient were analyzed in the subsequent results, and compared with patients without genetic variant. For detailed information, please refer to the following results. The detailed clinical characteristics of the 4 patients are shown in Table 1.

**Table 1:** Clinical characteristics and outcomes of 4 children with ANE.

Cases	P1	P2	P3	P4
Age	1.9	2.1	6	1.1
Sex	Male	Female	Female	Male
History	Healthy	Healthy	Febrile seizure	Healthy
Family history	No	No	No	No
Prodromic infection	Yes	Yes	Yes	Yes
Glasgow score	9	9	11	8
Disturbance of consciousness	Yes	Yes	Yes	Yes
Disturbance of language	Yes	Yes	Yes	Yes
paralysis	No	No	No	No
Brain affected area (cranial MRI)	Thalamus, basal ganglia	Thalamus, basal ganglia, brain stem, cerebellum, corpus callosum	Thalamus, cerebellum	Thalamus, basal ganglia
Cerebrospinal fluid				
Cell count 10^6^/L	7	1	2	5
Protein mg/L	917.5	1930	856	712
Genetic Test	Truncation variant in the CRMP2 gene	No	No	No
Prognosis	Death	Intellectual disability and refractory epilepsy	Disturbance of language	Intellectual disability and refractory

### Changes in serum immune cells in patients with ANE

Compared with the normal reference value, the absolute counts of T lymphocytes, helper T cells, suppressor T cells, and NK cells in three younger patients (P1, P2, P4) showed varying degrees of decrease, while B cells did not exhibit significant changes. Among them, the decrease in T cells was particularly significant in P1 patient (Table 2).

**Table 2:** Detection of serum immune cells in 4 children with ANE.

	P1 ^1^	P2 ^1^	P3 ^2^	P4 ^1^
Absolute value of T lymphocytes	**693.5↓**	938.9↓	1400.2	1114.5↓
Absolute value of B lymphocytes	460.2	588.8	873	839.1
Absolute number of helper T lymphocyte	456.7↓	593.6↓	749	759.5↓
Absolute number of cytotoxic T lymphoid cells	208.5↓	289.9↓	485↓	595.9↓
Absolute number of NK cells	**23.8**↓	158.6↓	115.5↓	190↓
Immunoglobulin (IgA, IgG, IgM)	Normal	Normal	Normal	Normal
Serum antinuclear antibody	Normal	Normal	Normal	Normal

^1^ Reference values for 1–4 years old: absolute values of T lymphocytes (1794-4247/μl), B lymphocytes (461–1456/μl), CD4 T cells (902–2253/μl), CD8 T cells (580–1735/μl), and NK cells (270–1053/μl); ^2^ Reference values for 4–8 years old: absolute values of T lymphocytes (1424–2664/μl), B lymphocytes (280–623/μl), CD4 T cells (686-1358/μl), CD8 T cells (518–1125/μl), NK cells (258–727/μl).

Bold indicates a more significant decrease in the value.

### Changes in CSF cytokines in patients with ANE

Compared to normal reference values, the levels of IL-1β, IL-5, IL-6, and IL-8 were significantly increased, with patient P1 showing significantly higher values than the other three patients (Table 3).

**Table 3:** Changes of cytokines in cerebrospinal fluid of children with ANE.

Cytokine	Reference value (pg/ml)	P1	P2	P3	P4
IL-1β	<12.4	88.5↑	34.2↑	57.9↑	67.1↑
IL-2	<7.5	2.3	3.1	4.2	13.4↑
IL-4	<8.56	1.05	0.97	1.02	0.88
IL-5	<3.1	40.2↑	49.4↑	33.2↑	50.9↑
IL-6	<5.4	253.1↑	99.5↑	185.3↑	12.6
IL-8	<20.6	1475.3↑	1283.1↑	974.3↑	716.9↑
IL-10	<12.9	1.11	0.97	1.02	2.01
IL-12p70	<3.4	6.24	7.11	4.74	7.12
IL-17	<21.4	2.36	6.36	7.12	3.93
IFN-α	<8.5	7.12	5.73	6.48	8.22
IFN-γ	<23.1	10.28	11.21	9.87	14.58
TNF-α	<16.5	3.6	6.1	5.2	4.6

### Genetic testing results of ANE patients

**Figure 1:**
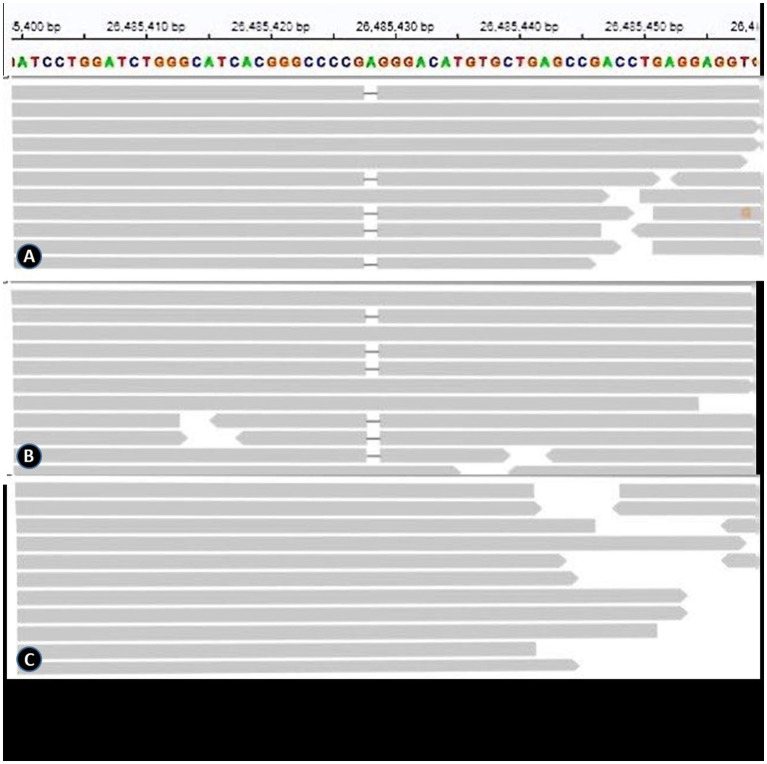
**(A)**, **(B)** and **(C)** represent the genetic results of the patient, the patient’s father, and the patient’s mother, respectively.

All gene variants identified through whole exome sequencing were screened using keywords such as epilepsy, ANE, and immune deficiency. No rare (<0.1gnomAD) or pathogenic (1 predicted harmful by software) variants were found in known ANE-related genes. However, it was found that patient P1 harbored a heterozygous truncation variant in the CRMP2 gene, which is related to T cell migration (NM_001197293.3: c.977del, Figure 1). This variant was confirmed to be of paternal origin by family verification. Due to the loss of follow-up, it remains uncertain whether the father had a medical history related to this variant. This gene is classified as a Probability of Loss of Function Intolerant (pLI > 0.95) gene, suggesting that this genetic variant may be related to the pathogenesis of ANE (see Figure 1).

## Discussion

ANE is a severe form of encephalopathy, characterized by bilateral symmetrical thalamic necrosis. ANE primarily affects previously healthy children and is associated with high mortality rates, and survivors, often leaving survivors with serious sequelae such as intellectual disability and refractory epilepsy. It is frequently triggered by respiratory virus infection, with common pathogens including influenza virus (10), novel coronavirus (11, 12), respiratory syncytial virus, parainfluenza virus and others. In a retrospective analysis of 983 cases of Acute Encephalopathy (AE) in Japan, Hoshino et al. identified influenza virus as the primary pathogen (13). Recent years have witnessed the emergence of COVID-19 as a prominent respiratory virus, with increasing attention paid to its neurological complications. Studies have shown that 2.2% of COVID-19-related infections are complicated with encephalopathy, resembling the neurological complications caused by influenza virus (14, 15). In this study, all four ANE patients had a precursor infection with the novel coronavirus, specifically the Omicron BF.7.14 variant, which is the first reported instance of this variant causing ANE in children.

The pathogenesis of ANE may be related to cytokine storms (16–18), characterized by elevated levels of neurotoxic cytokines such as IL-6 and TNF- α, which are known to damage to the blood–brain barrier, resulting in cytotoxic edema and vascular edema, petechial bleeding and necrosis (19). A previous study (20) in Japan showed that high serum IL-6 (>15,000 pg/mL) as a poor prognostic indicator for childhood ANE, associated with a 100% mortality rate. Our study found significant increases in IL-1 β、IL-5, IL-6, and IL-8levels, with IL-6 and IL-8 showing particularly significant elevations, indicating that the aforementioned cytokines are involved in the occurrence of this disease. Notably, the highest levels of IL-6 and IL-8 were observed in patient P1, whose prognosis was the most severe, highlighting a strong positive correlation between the severity of prognosis and these cytokine levels. Furthermore, we observed a significant decrease in the number of serum T cells and NK cells (prior to immune therapy), with P1 exhibiting a more substantial decrease. This phenomenon has not been reported yet. Previous studies have demonstrated increased cytokine levels, including TNF-α, IL-6, and IL-10, in patients with COVID-19 (21). Interestingly, these studies revealed a negative correlation between serum T cell counts and cytokine levels, suggesting that higher cytokine levels are associated with lower T cell counts. Genetic variant are the underlying genetic basis of ANE. Known ANE-related genes include RANBP2, CPT2, UNC93B1, NUP214, SNORA31, IRF3, TBK1, TICAM1, TRAF3, TLR3. A study in 2009 reported the presence of missense variant in the RANBP2 gene in familial or recurrent ANE cases and identified these variant as associated with ANE I. However, the author noted that RANBP2 gene missense variant have phenotypic penetrance differences, suggesting that not every variant carrier experiences ANE. Therefore, the author proposed that specific time point viral infection is another important environmental factor for variant carriers to develop ANE (4, 5). In addition, Shinohara et al. reported CPT II gene variant in ANE patients.

Although no pathogenic variant were found in the previously known ANE-related genes in the four children with ANE in our study, we identified a heterozygous truncation variant in the CRMP2 gene, associated with T cell migration in patient P1 (NM_001197293.3: c.977del).

Collapsin response mediator protein 2 (CRMP2) is a microtubule binding protein abundant in neural tissue. CRMP2 not only promotes stable microtubule assembly but also plays a role in cell migration, differentiation, protrusion growth, plasticity, and regeneration (22). Under normal circumstances, there are a small number of T cells in the central nervous system. In cases of inflammation, T cells are recruited to the inflammatory site. Recent studies have shown that CRMP2 is highly expressed in T lymphocytes, and increased CRMP2 expression enhances T lymphocyte migration, particularly in cases of neuroinflammation (23–25). When normal individuals suffer from neuroinflammation related diseases caused by human T cell leukemia virus-I (HTLV-I) infection, CRMP2 expression increases (ref). At the same time, T lymphocytes undergo migration, and CRMP2 plays an important role in activating T lymphocytes to exert anti-inflammatory effects, indicating that this gene plays an important role in nervous system inflammation. In addition, recent literature (26) has reported that the newly generated missense variant of the CRMP2 gene leads to intellectual disability during the development of central nervous system. Moreover, previous study (27) has shown that the CRMP2 gene causes neuronal death in Huntington’s disease (HD) by participating in mitochondrial morphology and motor ability. In summary, patient P1 carries a truncated variant in the CRMP2 gene, probably resulting in a significant decrease in T cell counts and a severe cytokine storm in the cerebrospinal fluid (CSF). These findings suggest that the truncated variant likely impacts the expression or migration of T cells, rendering them unable to play their anti-inflammatory role in the nervous system during COVID-19 infection, ultimately leading to fatal ANE. Although the phenotype information of the patient P1’s father is unknown, the possibility of a CRMP2 gene variant, akin to the phenotype variability seen with missense variant in the RANBP2 gene, cannot be excluded. We aspire to conduct further in-depth research on patients carrying such variant in the future.

The limitation of this study is the small sample size. And we need to conduct more basic research to clarify whether the genetic variation is pathogenic. In addition, COVID-19 can also cause asymmetric/mild symptoms of the central nervous system, even though elevation of neuroaxonal injury biomarkers (28).

In conclusion, this study reports the clinical phenotype and prognosis of ANE related to the Omicron BF.7.14 variant of COVID-19, and finds a new candidate gene related to ANE through genetic analysis. It is the first report of the CRMP2 gene’s involvement in ANE patients. Our findings suggest that this genetic variation may influence ANE prognosis by affecting T cell migration within the brain. This study opens up avenues for further research on novel genes in ANE and highlights the need for a deeper understanding of the disease.

## Data availability statement

The raw data supporting the conclusions of this article will be made available by the authors, without undue reservation.

## Ethics statement

The studies involving humans were approved by the Medical Ethics Committee of the Capital Institute of Pediatrics, with ethics number SHERLM2023061. The studies were conducted in accordance with the local legislation and institutional requirements. Written informed consent for participation in this study was provided by the participants’ legal guardians/next of kin. Written informed consent was obtained from the individual(s) for the publication of any potentially identifiable images or data included in this article.

## Author contributions

JZ: Writing – original draft. JS: Formal analysis, Writing – review & editing. DL: Data curation, Writing – review & editing. HX: Investigation, Writing – review & editing. SS: Data curation, Software, Writing – review & editing. YG: Data curation, Writing – review & editing. HJ: Formal analysis, Writing – review & editing. CM: Formal analysis, Writing – review & editing. ZL: Formal analysis, Writing – review & editing. LZ: Methodology, Writing – review & editing. JY: Project administration, Supervision, Writing – review & editing.
